# Thermomechanical characterization of bioplastic films produced using a combination of polylactic acid and bionano calcium carbonate

**DOI:** 10.1038/s41598-022-20004-1

**Published:** 2022-09-15

**Authors:** O. J. Gbadeyan, L. Z. Linganiso, N. Deenadayalu

**Affiliations:** 1 Department of Chemistry, Durban University of Technology, Steve Biko Campus, Durban, KwaZulu-Natal Province Republic of South Africa; 2 Honorary Research Associate, Faculty of Applied Sciences, Durban University of Technology, P O Box 1334, Durban, 4000 Republic of South Africa; 3 Green Engineering Research Focus Area, Faculty of Engineering and Built Environment, Durban University of Technology, Durban, Republic of South Africa

**Keywords:** Biochemistry, Biotechnology, Drug discovery, Environmental sciences, Environmental social sciences, Materials science, Nanoscience and technology

## Abstract

The present study focuses on the thermomechanical investigation of bioplastic firms produced from a combination of polylactic acid (PLA) and nano-calcium carbonated (nano-CaCO_3_) synthesized from the *Achatina Fulica* snail shell. The bioplastic films fabricated with nano-CaCO_3_ content ranging from 1 to 5 wt% were prepared using a solvent casting method. Thermal stability and degradation with temperature-dependent mechanical properties such as stiffness, storage modulus, and loss modulus of the developed bioplastic films were determined. The conformation changes in the functional group of the developed bioplastic films after incorporating nano-CaCO_3_ were also investigated. It was observed that incorporating nano-CaCO_3_ improved the thermal stability and temperature-dependent mechanical properties of neat PLA, regardless of the percentage weight added. An 85.67% improvement in thermal stability was observed. The temperature-dependent stiffness increased by 84%, whereas the storage modulus improved by 240%. On the other hand, loss modulus improved by 50% due to nano-CaCO_3_ incorporation into PLA. The FTIR curves of bioplastic films incorporated with nano-CaCO_3_ present insignificant conformation changes in the functional group of the resulting bioplastic films. This is presumable due to the compatibility of the matrix and the reinforcement. As a result, the resulting materials' thermal and temperature-dependent mechanical properties improved significantly, demonstrating that the developed bioplastic films could be used for package applications.

## Introduction

The demand for plastic-based packaging material has increased globally. The demand and excessive use of plastic have detrimentally affected the terrestrial and aquatic environment, leading to environmental pollution and the death of many aquatic animals^1,2^. The global market size for plastic packaging was valued at USD 348.08 billion in 2020 and is projected to increase by 4.2% from 2021 to 2028 on yearly compound bases^3^. This expected annual increase is due to the swiftly growing demand for plastic in several packaging applications in the agricultural and pharmaceutical sectors.

**Figure 1:**
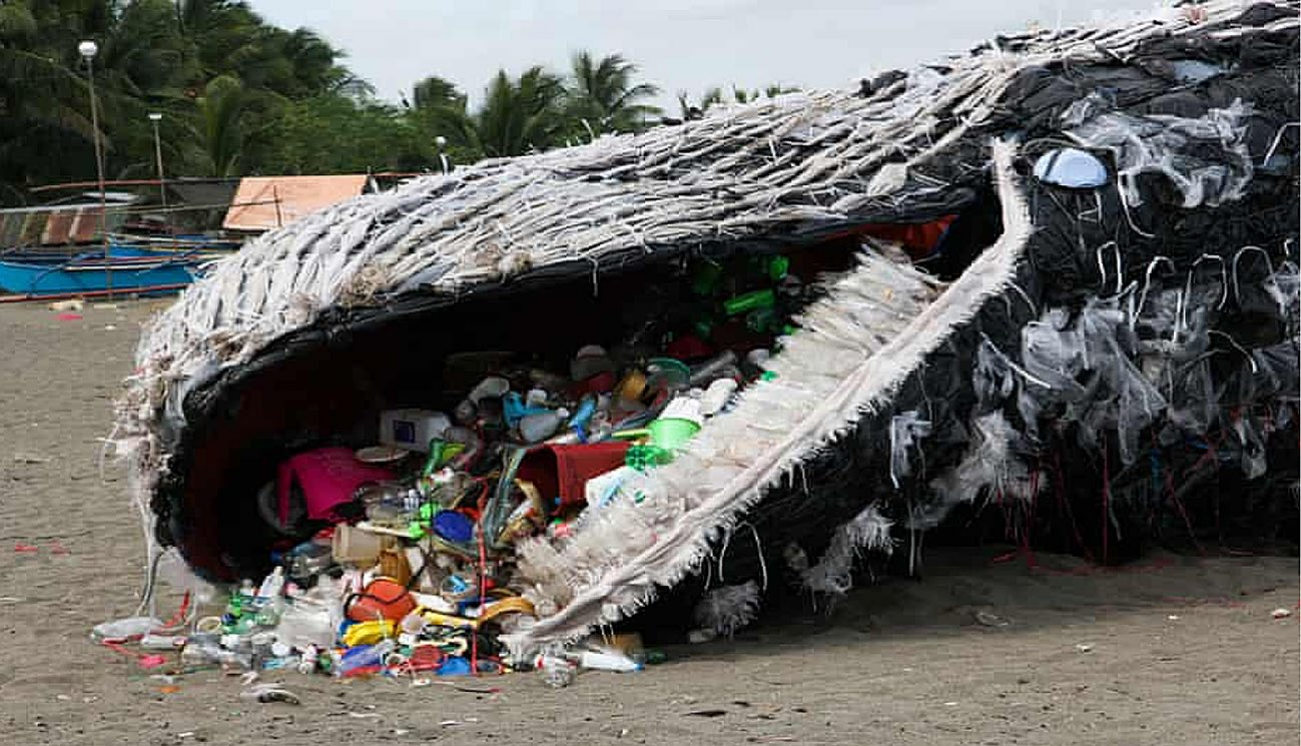
The whale swallowed plastic debris and died^2^.

In particular, the global demand for plastics in agriculture rose from 4.4 to 7.4 million tonnes from 2015-to 2019, presenting a 20% yearly increase^4–6^. The key drivers for increasing plastic consumption include but are not limited to: (1) Increasing population worldwide; (2) Plastics remain one of the world's most cost-effective materials with applications across every industry; (3) Recycling presents its shortcomings. However, the increased plastic demand corresponds to environmental pollution (Fig. 1), as readily available plastic is petroleum-based. As the population increases, plastic waste in landfill sites increases, leading to biodiversity loss and destroying our ecosystem (Fig. 1). Whales, some sharks, and other marine species are increasingly at risk from microplastics in the oceans. Plastic pollution is our biggest challenge. For example, plastics such as high-density polyethylene (HDPE), low-density polyethylene (LDPE), polypropylene (PP), and polystyrene (PS) are commonly used for packaging applications due to their lightweight, strength, affordability, and availability. However, these plastic materials are organic polymers produced from petroleum-based plastics. These synthetic plastics pollute our environment because of their excessive use. Also, their light weight allows them to travel long distances, resulting in extreme pollution on land and water^7,8^.

Furthermore, the increase in plastic demand and the availability of organic polymers, the available plastic materials may not meet the need for plastic in decades to come. As a result, alternative solutions are being sought to mitigate the shortcomings mentioned above. Extensive research is being undertaken to produce plastics from renewable sources.

Several efforts have been taken toward reducing the severe effects caused by use crude oil derived plastic leading to research developing biobased plastics for a combination of different biomass and naturally sourced nanoparticles^9–13^. Plastic materials such as PVA^14,15^, Polycarbonate^16^, and PLA have been explored for bioplastic film development due to their availability, cost-efficient, and non-petroleum. Similarly, the development of PLA showcases a bioplastic polymer that is non-toxic, biodegradable, has better strength and modulus with good film-forming characteristics, and could be used for short lifespan applications^14^. These characteristics increased their uses in food packaging and drug delivery purposes. However, the strength of plastic, such as PLA, dropped when exposed to heat because of its poor thermal resistance properties, which suggested reinforcing PLA with nanofillers^17–20^.

Over the decades, researchers have explored reinforcing bioplastic with different nanofiller and biobased fillers^21–24^. The study revealed that adding biobased nanoparticle reinforcement is a viable way of improving bioplastic strength^20,25,26^. Carbon-based materials such as calcium carbonate CaCO_3_ and cellulose of different sizes produced from sources such as agriculture and animal waste have been explored^9–13^. However, many nanomaterials are yet to be synthesized from biomass, the latter being considered waste bio-based nanomaterials, and some are considered waste. The material of choice used as a reinforcement in this study is underutilized and dumped for landfill purposes. However, biomass could serve as nanofiller feedstock for developing bio-based and biodegradable plastic materials^27–30^. Despite the rigorous improvement of bioplastic film material development, nano-CaCO_3_ was synthesized from discarded *Achatina Fulica* snail shells, and polylactic acid has not been combined for bioplastic films. In this regard, in this study, nano-CaCO_3_ was synthesized from discarded *Achatina Fulica* snail shell, and polylactic acid (PLA) was combined to produce robust bioplastic films. The bioplastic thermomechanical properties were investigated to determine thermal stability, degradation, and temperature-dependent mechanical properties such as storage modulus, loss modulus, and glass transition temperature.

## Experimental details

Polylactic acid Ingeo Biopolymer 10361D (granular) purchased from Sigma-Aldrich USA was used as a binder. Nano-CaCO_3_ with particle sizes ranging from 25 to 63.68 nm produced from *Achatina Fulica* shell through mechanochemical techniques (ball milling) described in our previous study were used as filler^31^. Acetone used for dissolving polylactic granular was supplied by Laboratory and Analytical supplies (PTY) limited Durban.

### Preparation of Nano-CaCO_3_ /PLA bioplastic film

**Figure 2:**
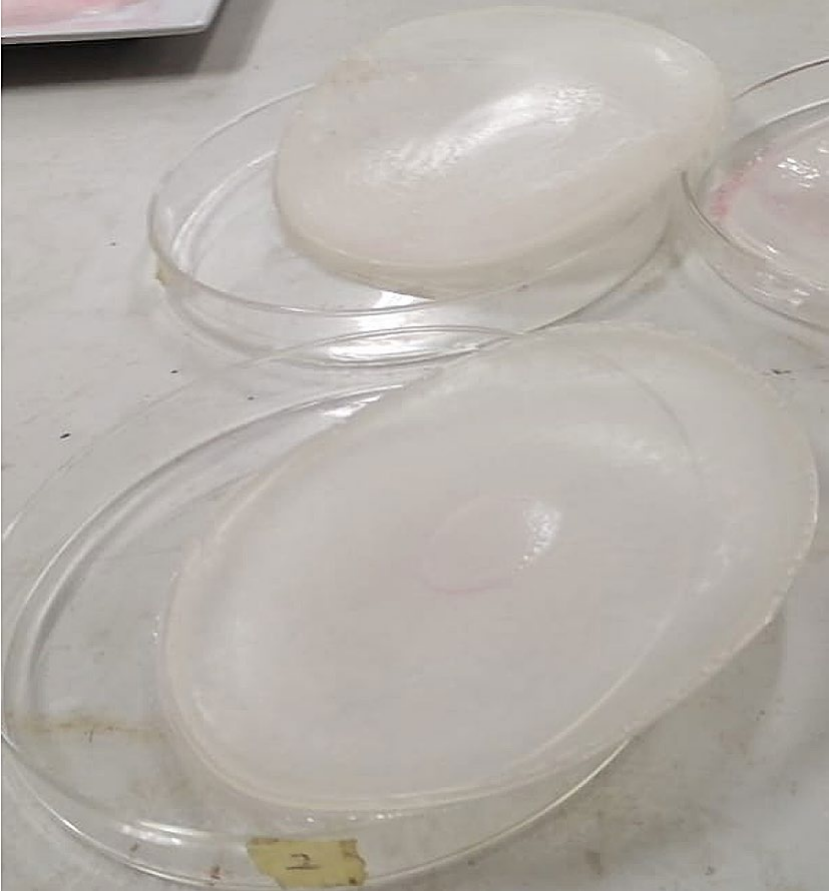
Nano-calcium carbonate reinforced polylactic acid films.

Bioplastic films containing nano-CaCO_3_ and PLA were developed using the solvent casting method. The technique was achieved in four steps. The drying of polylactic granular to remove retaining moisture was the first step, and the second step involved the dissolution of polylactic granular in acetone. The third step was the dispersion of the nano-CaCO_3_ in the polylactic/acetone solution, and the fourth step entailed casting and de-molding of bioplastic films. In the first step, 90 g of polylactic granular were measured into a beaker and kept in a vacuum oven at 60 °C for 30 min to eliminate moisture. Well-dried polylactic granular were then removed and dissolved in acetone using a dissolution ratio of 1 g polylactic granular to 3 ml acetone. The pre-calculated acetone and polylactic granular dissolution portions were measured in a glass container. This container was covered for 72 h with a fitting lid. After that, the polylactic/acetone solution was stirred at 500 rpm using a mechanical stirrer to ensure the complete dissolution of polylactic acid. The polylactic solution was allowed to settle to prevent bubbles that cause porosity in plastic materials. Then, the PLA and nano-CaCO_3_ solution of different loading ratios (1–5% wt) were combined in a beaker and stirred at 400 rpm for 60 min using a magnetic stirrer to ensure uniform dispersion_._ Subsequently, the blend was poured into a glass mold and allowed to cure for 24 h at ambient temperature. The thermomechanical properties of the developed bioplastic films shown in Fig. 2 were determined after 15 days to ensure adequate curing.

### Fourier transform infrared spectroscopy

The effect of loading different concentrations of nano-CaCO_3_ on functional groups of bioplastic films was analyzed using Fourier transform infrared spectroscopy (FTIR). A universal attenuated total reflectance module was used for the analyzed bioplastic film samples' spectra in a wavenumber range between 4000 and 600 cm^−1^.

### Thermal properties

Bioplastic film thermal stability and degradation were concurrently investigated on a thermal gravimetric analyzer (TGA)(Thermal Universal V 4.5 A). This thermogravimetric analysis was performed at a 10 °C/min heating rate under a dry nitrogen gas flow at 100 mL/min from 30° to 600 °C.

### Dynamic mechanical analysis

Temperature-dependent stiffness, storage modulus, and loss modulus of the developed nano-CaCO_3_ reinforced polylactic acid bioplastic films were investigated using a dynamic mechanical analyzer (DMA). This measurement was obtained on 60 mm × 1.2 mm × 1.25 mm bioplastic film specimen size at a frequency of 100 Hz in a 3-point bending mode (TA instruments model Q800) from 20 to 100 °C under atmospheric conditions. The data generated by the dynamic mechanical analyser was used for graphical illustration.

## Results and discussion

### Thermal behavior

**Figure 3:**
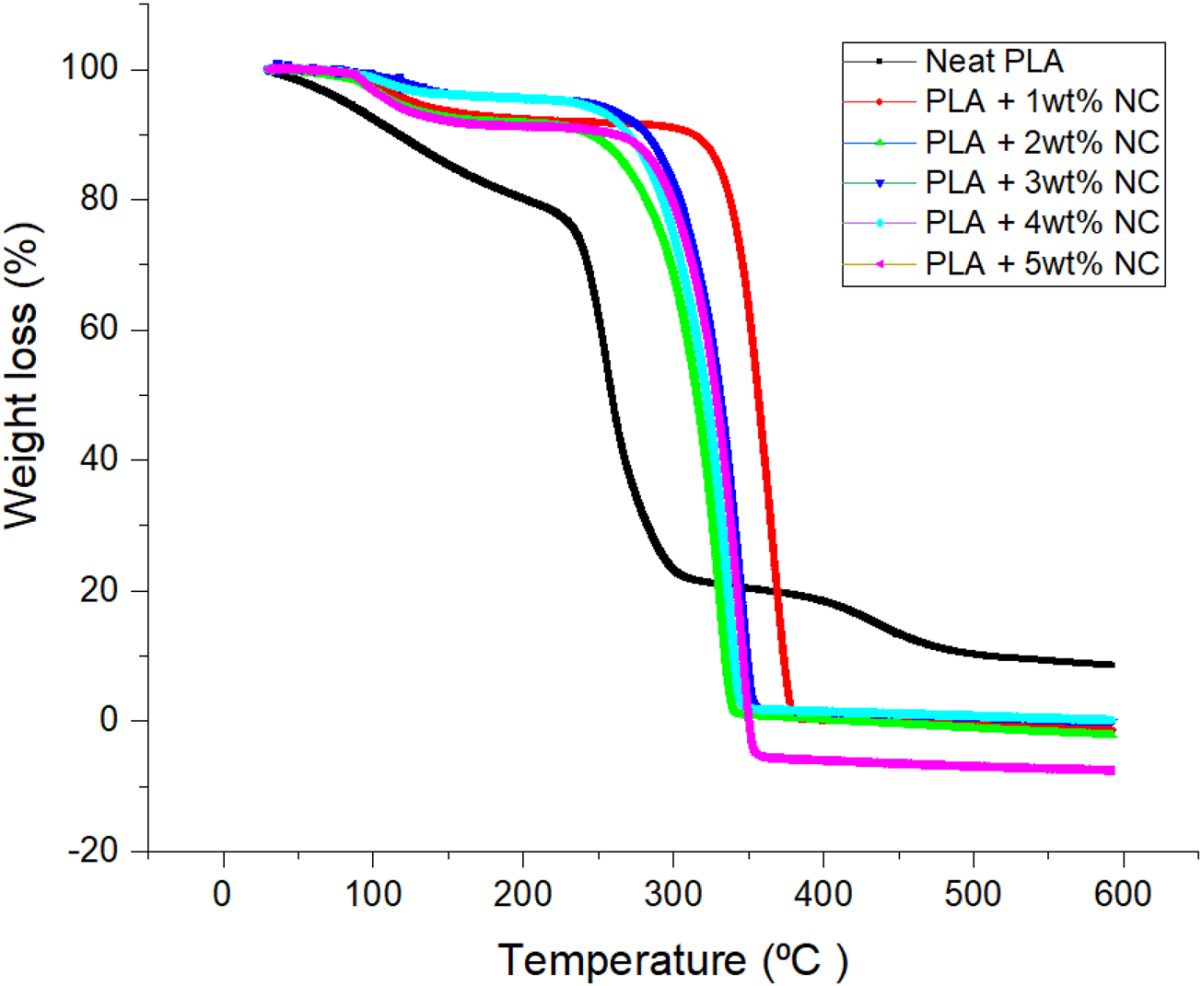
TGA thermogram curve of unfilled and nano-CaCO_3_ reinforced polylactic acid bioplastic films.

As shown in terms of thermal stability and degradation, Fig. 3 presents the thermal behavior of pure PLA and nano-CaCO_3_ reinforced bioplastic films. The addition of nano-CaCO_3_ significantly enhanced the temperature regions of PLA. Bioplastic films with nano-CaCO_3_ lose moisture at temperatures up to 100 °C. Afterward, two decomposition temperatures were observed for nano-CaCO_3_ reinforced PLA bioplastic films. Three thermal events were observed for neat PLA and bioplastic with nano-CaCO_3,_ show two phases. The first phase was seen at a temperature between 281 and 342 °C, proving variance in degradation temperature of these bioplastic series with corresponding loading percentage. The degradation can be attributed to the fragmentation of organic materials such as nano-CaCO_3_ in the bioplastic films^32^. Although incorporating nano-CaCO_3_ improved thermal stability, it was observed that the thermal stability varied with different percentage loading of the nano-CaCO_3._ Bioplastic films with 1wt% exhibited thermal stability, which was 51% better than neat PLA. Figure 3 shows that thermal property of neat PLA stable upto 225 °C, while thermal property of bioplastic films with 1wt% stable upto 340 °C.

This thermal output can be directly attributed to the effectiveness of loading a small amount of nano-CaCO_3._ Similarly, uniform dispersion with the excellent thermal property of the filler material is another reason for the increased thermal behavior observed^33^. It may also be attributed to nano-CaCO_3_ disintegration of organic materials, which is the nano-CaCO_3_ incorporated into bioplastic films^32^. These results correspond with literature when the incorporation of naturally sourced nano-CaCO_3_ improves the thermal properties of composite materials^34–36^.

The thermal event seen between 250–600 °C is the second phase for neat PLA. Although carbon dioxide decomposed to calcium oxide (ashes) in the third phase between 340–600 °C, insignificant weight loss was observed. These organic materials disintegrations resulted in a weight loss in this phase equated to 85.67%. This performance implies that the nano-CaCO_3_ in the bioplastic films was responsible for increasing the thermal stability temperature of PLA from 198 to 340 °C. This thermal performance can be attributed to the inherent thermal property of the incorporated nanoparticle^27,37^.

### Fourier transform infrared spectrometry

**Figure 4:**
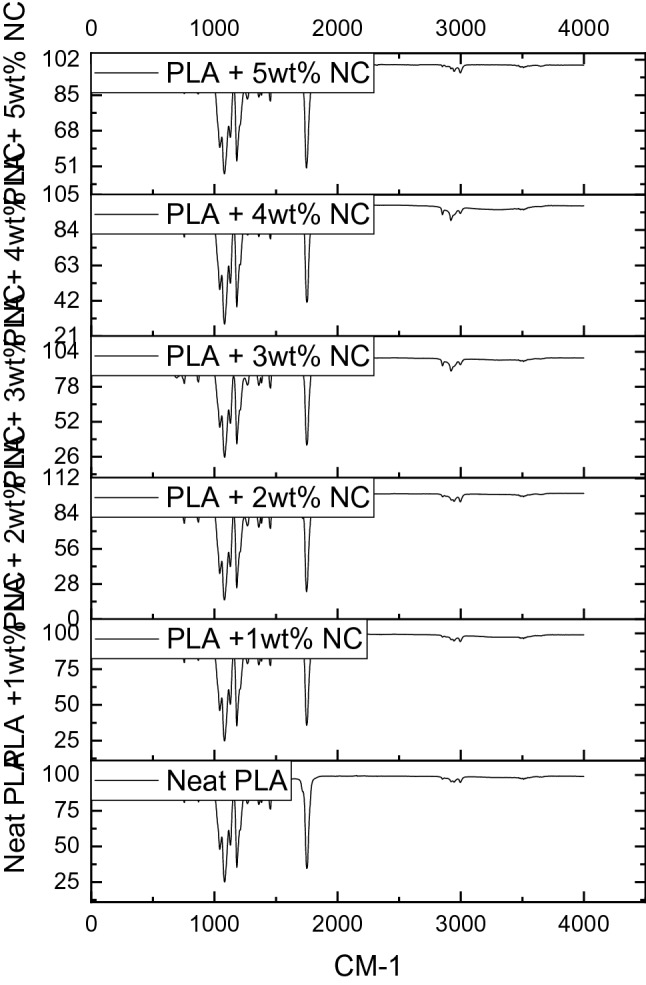
Fourier transforms infrared (FTIR) spectra of unfilled and nano-CaCO_3_ reinforced polylactic acid bioplastic films.

Fourier transform infrared (FTIR) spectrum was conducted on unfilled and nano-CaCO_3_ reinforced polylactic acid bioplastic films to investigate conformation changes in the functional group of bioplastic films. As shown in Fig. 4, numerous peaks were observed within 600–4000 cm^−1^. However, the peak becomes visible and shapers between 1000 and 4000 cm^−1^, confirming the semi-crystalline of the bioplastic films. This trend corresponds to Paragkumar et al. report^38^, in which sharp FTIR peaks are related to semi-crystallinity.

These high-pitched peaks dominated spectra of bioplastic films with different loading nano-CaCO_3_ produced from *Achatina Fulica* shell, indicating that the developed films are polylactic acid-based. The FTIR curves of bioplastic films with nano-CaCO_3_ showed insignificant conformation changes in the functional group, attributed to the compatibility of the matrix and the reinforcement. Due to the O–H stretching, a strong, broad intermolecular bonding at 3509 cm^−1^ was observed. An O–H stretching absorption peak relates to the prepolymer of snail shell nanoparticles and PLA elemental composition^39^. This absorption peak may be attributed to intra- and extra-molecular hydrogen bonding from the covalent bonds due to the transfer of atoms between matrix and nanofiller forming catenation^21^. The peak at 2919 cm^−1^ and 2944 cm^−1^ was associated with the C–H stretching vibration of alkane, presenting higher bonds to heavier atoms.

Furthermore, these bands may be attributed to the asymmetric and symmetric vibration stretching of aliphatic C-H, implying that bioplastic films contain carbon and hydrogen together in straight chains. Moreover, a very projecting absorption peak was observed at 1357 cm^−1^, related to OH bending carboxylic acid, showing carbon and oxygen in polylactic acid. Furthermore, two prominent absorption peaks were observed at 1080 cm^−1^ and 1788 cm^−1^ associated with C–O stretching, respectively. The C–O stretching at 1080 cm^−1^ is the primary acetone linked to the solvent used to dissolve PLA. At the same time, the C–O stretching at 1788 cm^−1^ is associated with conjugated solid alkenes in the film.

The absorption peaks observed at 1042 cm^−1^, 916.92 cm^−1^, and 842 cm^−1^ were associated with carbonate ions due to nano-CaCO_3_ in the film^25^. Most of the spectrum displayed a leading band that indicates the interaction between carbon and single-bonded hydroxyl bending in carboxylic acids and carbon single-bonded oxygen stretching in-plane carbon, indicating the functional group of the bioplastic film developed.

### Dynamic mechanical analysis

**Figure 5:**
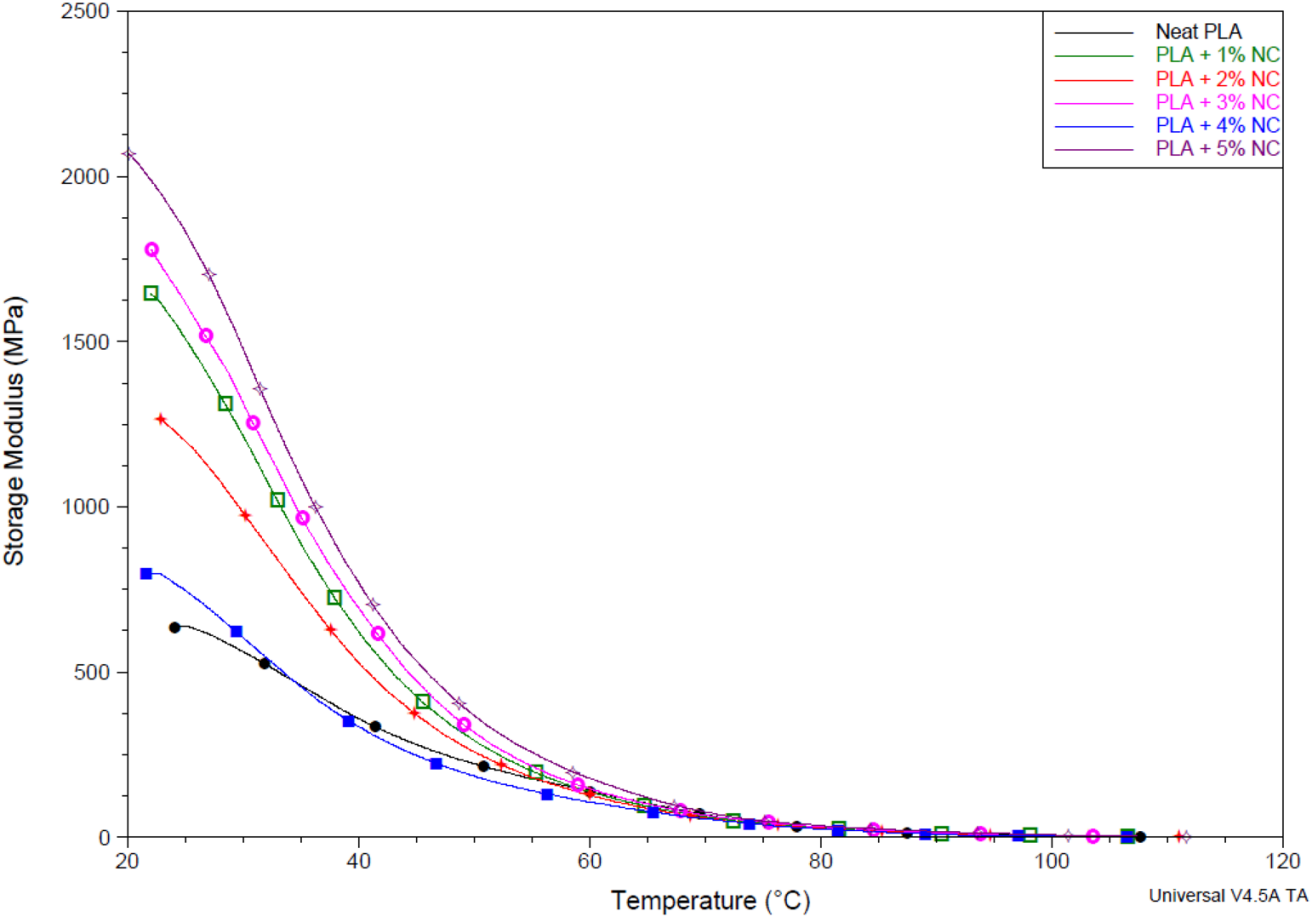
Storage modulus unfilled and nano-CaCO_3_ reinforced polylactic acid bioplastic films.

**Figure 6:**
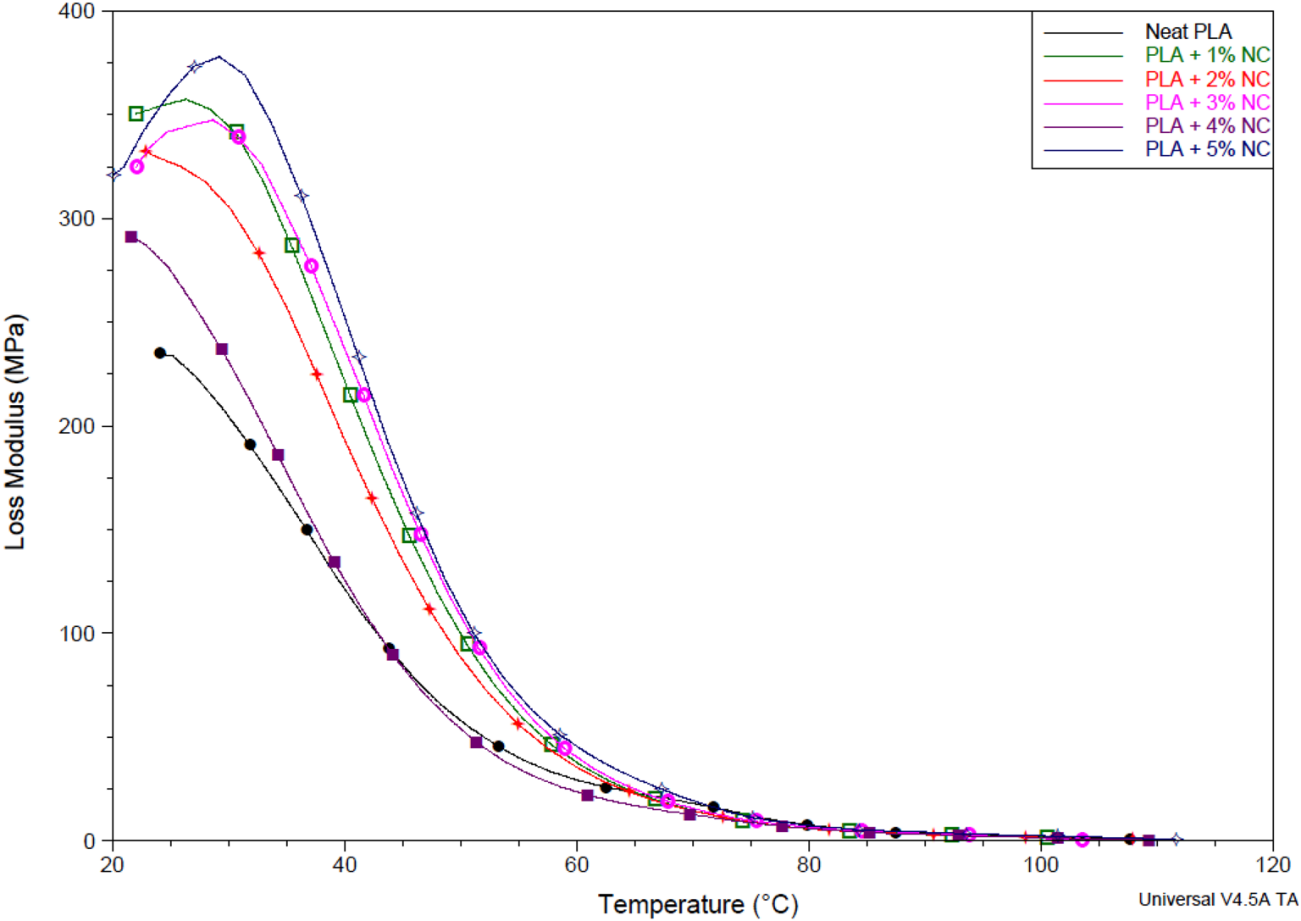
Loss modulus of unfilled and nano-CaCO_3_ reinforced polylactic acid bioplastic films.

**Figure 7:**
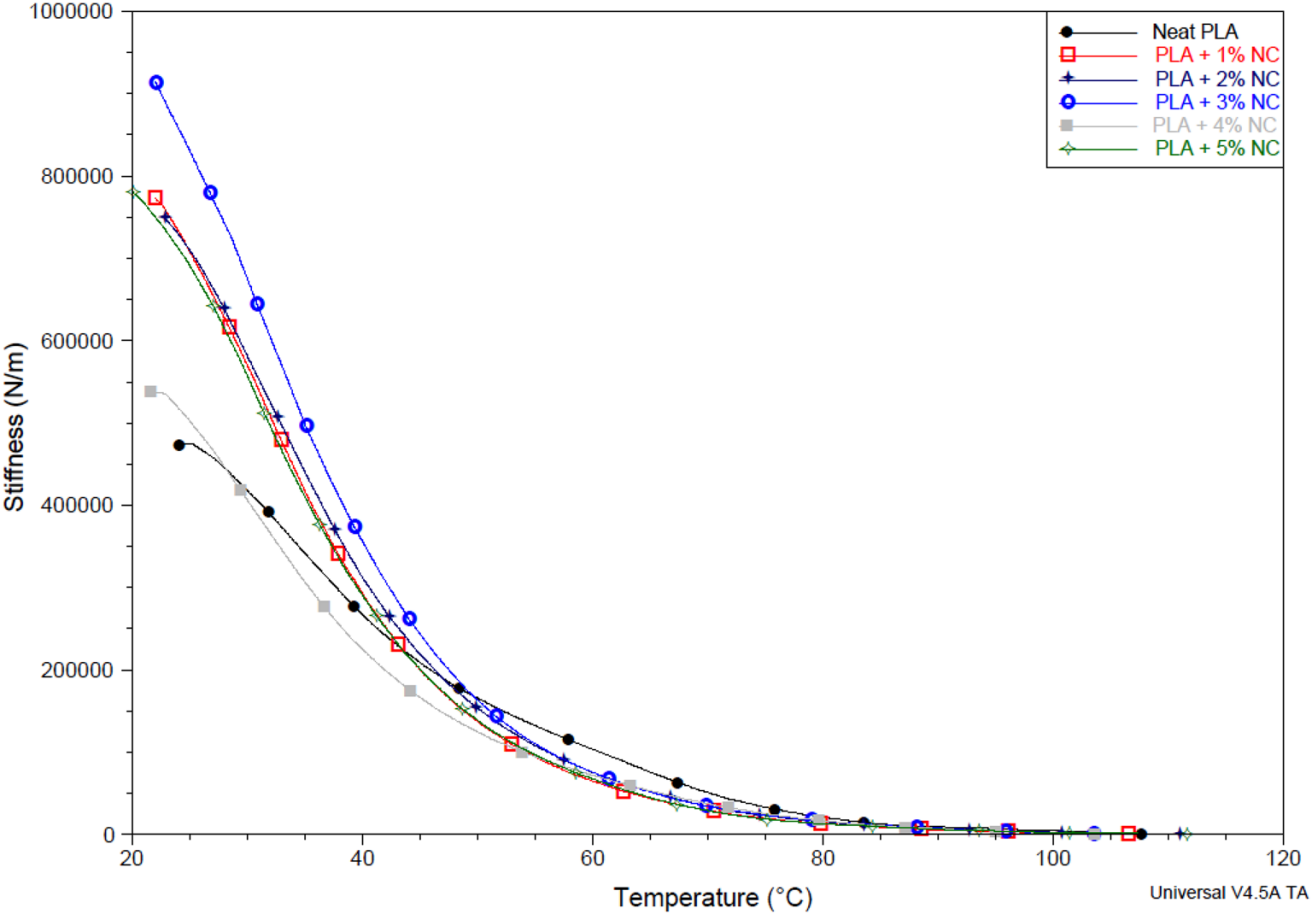
Stiffness of unfilled and nano-CaCO_3_ reinforced polylactic acid bioplastic films.

Temperature dependence modulus and stiffness of bioplastic films were determined in tensile mode using a dynamic mechanical analyzer reported in Figs. 5, 6 and 7. It was discovered that films exhibited different temperature dependence storage modulus (E'), and loss modulus (E"), stiffness with different nano-CaCO_3_ loading. This performance pronounces the effectiveness of nano-CaCO_3_ loading on temperature dependence mechanical of the developed bioplastic firms, which corresponds with the Fig. 3 graph where loading of nano-CaCO_3_ improves the thermal stability of bioplastic films. It was observed that incorporating nano-CaCO_3_ increased temperature dependence storage modulus (E') and bioplastic film irrespective of loading. Figures 5 show a decrease in PLA storage modulus with a corresponding increase in temperature, and a drastic decrease in E' was observed in the rubbery region. The degree of freedom at the atomic level, the dilution of PLA molecules, resulted from the free molecular mobility of the polymer chains. This free movement of the polymer chain broke the cross-linking between the molecular chains, reducing storage modulus. However, adding nano-CaCO_3_ strengthened bioplastic films at the rubbery region at 20, increasing temperature dependence storage modulus. It was observed that the storage modulus increased with a corresponding increase in nano-CaCO_3_ concentration. This trend corresponds with studies on loading nano-CaCO_3_ temperature dependence storage modulus^36,40–42^.

The addition of nano-CaCO_3_ may have reduced polymeric chain movement due to its more formidable and temperature resistance ability than PLA, modifying bioplastic films and improving E′^40,41^. The increase in temperature dependence storage modulus may also be attributed to the synergistic effect formed with PLA and nano-CaCO_3_ forming a robust structure, enhancing thermal stress resistance that enhances E′^41,43^.

The temperature dependence loss modulus of bioplastic films is shown in Fig. 6. It was discovered that PLA exhibited decreased dissipating heat energy due to limited viscous motions. This performance may be due to the poor heat resistance of PLA. However, the incorporation of nano-CaCO_3_ of different concentrations increased PLA melt viscosity. This performation may be related to the excellent thermal properties of incorporated nanofiller, increasing heat dissipation, and absorption of the developed bioplastic films irrespective of loading percentages. Significantly, the loading of 1 wt% nano-CaCO_3_ increases the intensity of E" at the lowest processing temperature, indicating an increase in melt viscosity of the bioplastic films. This thermal occurrence can be ascribed to the high loading of the nanofiller_,_ resulting in an increased heat energy dissipation achieved due to the motion of viscous motions within the blend. Adding 1 wt% nano-CaCO_3_ increased the bioplastic thickened blend, reducing the viscous motion by replacing polymer–polymer hydrogen bonding with plasticizer hydrogen bonding forming strong covalent bonding that increases heat dissipation^42,44^.

Furthermore, the bonding formation improves PLA's workability and processability, which reduces viscosity at the processing temperature. Moreover, a shift in the bioplastic processing temperature and E" was observed with a corresponding increase in nano-CaCO_3_ incorporation. However, the shift in processing temperature was observed after loading 3 wt% nano-CaCO_3_ loadings, showing an improved shift in processing temperature better than bioplastic with 1 wt% nano-CaCO_3_. The increase in heat dissipation and shift in processing temperature may be attributed to the homogeneous dispersion of the grain and the effectiveness of nano-CaCO_3_ as low concentration loading. This thermal performance proved that nano-CaCO_3_ could increase the viscosity of PLA compositions by acting as excellent plasticizers^35,36^.

Figure 7 shows that the stiffness varies with temperature for unfilled and nano-CaCO_3_ reinforced polylactic acid bioplastic films. A decreased stiffness with a corresponding increase in temperature loading speed was noted. Bioplastic film stiffness resistance to external thermal stress under variable loading rates and temperatures provided its visco-elastic time-dependent behavior. The neat PLA exhibited the lowest visco-elastic under varying temperatures slightly above 20 °C. This performance may be attributed to breaking in the bond within PLA molecules. The softness of PLA could be another reason for the poor resistance to temperature dependence stresses observed. However, improved resistance to heat stress was observed after incorporating nano-CaCO_3_. Bioplastic films with nano-CaCO_3_ exhibited a significant margin of 84.2% higher than neat PLA between 20 and 40 °C. The bioplastic films with 3 wt% exhibited superior visco-elastic time-dependent behavior, followed by firms with 1 wt%, which correspond with the trend observed in Fig. 6. This performance can be attributed to the inherent stiffness of the incorporated nanoparticles. The interfacial bonding due to the transfer of atoms forming a formidable structure delays the effect of temperature stress, resulting in an improved temperature-dependent stiffness.

## Conclusion

Bioplastic film was successfully developed using a combination of polylactic acid and nano-calcium carbonate (CaCO_3_) synthesized from the *Achatina Fulica* snail shell. The bioplastic films with various nano-calcium carbonate (CaCO_3_) loading (1–5 wt%) were prepared using solvent casting. Its conformation changes in the functional group, thermal stability, and degradation with temperature-dependent mechanical properties such as stiffness, storage modulus, and loss modulus were investigated. The results show that incorporating nano-CaCO_3_ enhances thermal stability and degradation with temperature-dependent mechanical properties such as stiffness, storage modulus, and loss modulus, irrespective of loading volume. The interfacial bonding due to the transfer of atoms forming a formidable structure delays the effect of temperature stress, resulting in improved thermal and temperature-dependent mechanical properties. An insignificant conformation change in the functional group of the developed bioplastic films after incorporating nano-CaCO_3_ was observed. This performance was attributed to the compatibility of the matrix and nanofillers, which are naturally sourced. Although bioplastic films with nano-CaCO_3_ exhibited improved properties than pure polylactic acid films, the bioplastic film with 1 wt% exhibited superior properties. These improved properties suggest bioplastic film for several packaging applications, including but not food, drugs, and other goods packaging. Commercial production of the bioplastic will help to meet the demand for packaging.

## Data Availability

The datasets generated and analyzed during the current study are not publicly available because they violate the rules of the affiliated institution but are available from the corresponding author on reasonable request.
